# Detection of SARS-CoV-2 Variants via Different Diagnostics Assays Based on Single-Nucleotide Polymorphism Analysis

**DOI:** 10.3390/diagnostics13091573

**Published:** 2023-04-27

**Authors:** Eliana Specchiarello, Giulia Matusali, Fabrizio Carletti, Cesare Ernesto Maria Gruber, Lavinia Fabeni, Claudia Minosse, Emanuela Giombini, Martina Rueca, Fabrizio Maggi, Alessandra Amendola, Anna Rosa Garbuglia

**Affiliations:** Laboratory of Virology, National Institute for Infectious Diseases (INMI) Lazzaro Spallanzani (IRCCS), 00149 Rome, Italy

**Keywords:** SARS-CoV-2, molecular diagnosis, SARS-CoV-2 variants, single-nucleotide polymorphism (SNP), SARS-CoV-2 variant assay

## Abstract

Severe acute respiratory syndrome coronavirus 2 (SARS-CoV-2) is characterized by fast evolution with the appearance of several variants. Next-Generation Sequencing (NGS) technology is considered the gold standard for monitoring known and new SARS-CoV-2 variants. However, the complexity of this technology renders this approach impracticable in laboratories located in areas with limited resources. We analyzed the capability of the ThermoFisher TaqPath COVID-19 RT-PCR (TaqPath) and the Seegene Novaplex SARS-CoV-2 Variant assay (Novaplex) to detect Omicron variants; the Allplex VariantII (Allplex) was also evaluated for Delta variants. Sanger sequencing (SaS) was the reference method. The results obtained with *n* = 355 nasopharyngeal samples were: negative with TaqPath, although positive with other qualitative molecular assays (*n* = 35); undetermined (*n* = 40) with both the assays; negative for the ∆69/70 mutation and confirmed as the Delta variant via SaS (*n* = 100); positive for ∆69/70 and confirmed as Omicron BA.1 via SaS (*n* = 80); negative for ∆69/70 and typed as Omicron BA.2 via SaS (*n* = 80). Novaplex typed 27.5% of samples as undetermined with TaqPath, 11.4% of samples as negative with TaqPath, and confirmed 100% of samples were Omicron subtypes. In total, 99/100 samples were confirmed as the Delta variant with Allplex with a positive per cent agreement (PPA) of 98% compared to SaS. As undermined samples with Novaplex showed RdRp median Ct values (Ct = 35.4) statistically higher than those of typed samples (median Ct value = 22.0; *p* < 0.0001, Mann–Whitney test), the inability to establish SARS-CoV-2 variants was probably linked to the low viral load. No amplification was obtained with SaS among all 35 negative TaqPath samples. Overall, 20% of samples which were typed as negative or undetermined with TaqPath, and among them, twelve were not typed even by SaS, but they were instead correctly identified with Novaplex. Although full-genome sequencing remains the elected method to characterize new strains, our data show the high ability of a SNP-based assay to identify VOCs, also resolving samples typed as undetermined with TaqPath.

## 1. Introduction

Severe acute respiratory syndrome coronavirus 2 (SARS-CoV-2) is a member of the beta-coronavirus genus which was classified as a pandemic by the Word Health Organization (WHO) in March 2020 [1]. Like other RNA viruses, SARS-CoV-2 is characterized by an error-prone RNA-dependent RNA polymerase, which promotes the accumulation of mutations in a short period of time in addition to the recombination phenomenon between divergent strains, thus increasing genetic variability [2]. Specific sites of ORF1a, 1b, 3a, 8, N, and S genes are found under positive selection, allowing many variants to arise, with each being characterized by a specific set of mutations [3]. The Center for Disease Control and Prevention (CDC) has classified SARS-CoV-2 variants into four categories: variants of interest (VOIs), variants of concern (VOCs), variants of high consequence (VHCs), and variants being monitored (VBMs). All over the world, several VBMs circulate, but they are not considered a threat to public health; on the contrary, VOCs are deemed a threat to public health because of their high transmissibility, variability, ability to evade vaccination-induced immunity, and severe course of the disease. Six main VOCs have been reported: Alpha (B.1.1.7), Beta (B.1.351), Gamma (P.1), Delta (B.1.617.2) [4], and Omicron [5]. The Omicron variant, detected for the first time in November 2021 in Botswana and South Africa [5], shows high transmissibility and a high rate of mutation (in a few months, three Pangolin sublineages had been identified: BA.1, BA.2, and BA.3), and its evolution is still ongoing [6]. In fact, new variants have emerged (designated as BA.4 and BA.5), and they quickly spread in South Africa, Europe, and the United States [7]. The continuous surveillance of circulating SARS-CoV-2 variants is critical for molecular epidemiological surveillance, tracking the local emergence of new strains, and providing health authorities with the information they need to implement public health measures to reduce infection spread.

Whole-genome sequencing via Next-Generation Sequencing (NGS) technology is considered the gold standard for identifying known and new SARS-CoV-2 variants. However, this method has several limitations: the moderate number of specimens that can be processed, the complexity of technology, the specific and expensive equipment required, the high level of expertise necessary for data analysis, and the long turnaround time. Thus, alternative, cheaper technologies which are less labor-intensive, allow the easy interpretation of results, and have rapid and efficient workflows, allowing SARS-CoV-2 variant screening in low-resource laboratories, are needed. Moreover, the rapid identification of variants is essential for the administration of monoclonal antibody therapy, due to the lack of efficacy of certain antibodies against specific variants [8].

In addition to the NGS, alternative technologies, such as mass spectrometry [9] and CRISPR-based detection [10], allow the typing of SARS-CoV-2 variants, but the most commonly used and feasible approach is analysis of the single-nucleotide polymorphisms (SNPs) in spike protein-encoding genes via real-time RT-PCR. This method is cost-effective, faster than Sanger sequencing analysis (SaS) and produces results with precision comparable to that obtained via NGS [11,12]. Furthermore, the instruments’ ease of use and the lack of skills required for test execution make this assay suitable for use in any laboratory.

In this study, we compared the performances of two commercially available diagnostic assays, both based on the SNP method: the ThermoFisher TaqPath COVID-19 RT-PCR (TaqPath) and the Seegene Novaplex SARS-CoV-2 Variant VII assay (Novaplex) and Seegene Allplex variants II (Allplex). TaqPath is a real-time RT-PCR diagnostic kit that identifies the 69/70 deletion (∆69/70) in the Spike (S) gene, allowing one to distinguish SARS-CoV-2 variants [13]. Novaplex is a multiplex RT-PCR, and it is able to type Omicron Ba.1 and B.2 variants, while Allplex is used to identify Delta variants (B.1.617.2).

## 2. Materials and Methods

### 2.1. Sample Collection

Between December 2021 and March 2022, all nasopharyngeal swabs (NPSs) received in our laboratory were analyzed for SARS-CoV-2 with qualitative diagnostic molecular assays able to detect all viral variants circulating at that time (Aptima SARS-CoV-2 assay (Hologic Inc., Marlborough, MA, USA) or Abbott RealTime SARS-CoV-2 (Abbott Inc., Chicago, IL, USA)), as declared by the companies. Samples that were positive for SARS-CoV-2 were tested using TaqPath for rapid discrimination between the Omicron BA.1 variant and Delta variant and between the Omicron BA.1 variant and Omicron BA.2 variant, as recommended by the ECDC and WHO [14,15]. Several samples (see below) were then analyzed with the Novaplex SARS-CoV-2 Variant VII assay.

All procedures were approved by the Ethical Committee of INMI Lazzaro Spallanzani (n.70/2018).

### 2.2. RNA Extraction Protocol

All NPSs stored in universal transport medium were digested with proteinase K for 10 min at 56 °C prior to extraction. Total viral nucleic acid was extracted from 400 µL of each sample and eluted in a final volume of 60 µL using QIASYMPHONY instruments (QIAGEN, Hilden, Germany) according to the manufacturer’s instructions. Each extracted viral RNA was stored at −80 °C until use.

### 2.3. Molecular Assays for SARS-CoV-2 Variant Identification

SARS-CoV-2 variant identification was performed on viral nucleic acid extracts obtained from NPS as described above (RNA extraction protocol). In all runs performed for the typing of variant viruses, viral nucleic acid extracts obtained from positive samples already typed via Sanger sequencing as Delta, Omicron1 (BA.1), Omicron2 (BA.2), and Alpha variants were included as controls. SaS was considered as the reference method.

#### 2.3.1. TaqPath™ COVID 19 CE IVD RT PCR

TaqPath™ COVID 19 CE IVD RT PCR (TaqPath; ThermoFisher Scientific, Waltham, MA, USA) is a multiplexed RT-PCR assay based on three primer/probe sets specific to the ORF1ab, N gene, and S gene of SARS-CoV-2. The LLoD is 10 genomic copy equivalent/reaction (GCE/reaction) for both NPS and bronchoalveolar lavage specimens. The cycle threshold (Ct) cut-off value for the clinical target is ≤37 (according to TaqPath™ COVID 19 CE IVD RT PCR Instructions for Use). Although it was developed for the diagnosis of SARS-CoV-2 infections, this assay was used as a fast-screening method for variants of the new coronavirus [16]. In fact, in the presence of ORF1ab (detected) and N (detected) gene amplifications, samples showing the ∆69–70 S gene mutation resulted in S gene dropout (i.e., the S gene was not detected); this combination of results in the same sample was accordingly indicated as S gene target failure (SGTF). As this mutation was useful in identifying some SARS-CoV-2 variants, namely Alpha and Omicron BA.1, TaqPath was used here to discriminate SARS-CoV-2 variants in positive samples, in accordance with ECDC and WHO guidelines released at the beginning of December 2021. Samples identified as SGTF underwent SaS of the S gene (as described below). Since samples with high Ct values may show a pattern of SGTF by chance with a weak signal in the other targets as well, NPSs showing Ct values >32 for ORF1ab or the N gene in the absence of S gene amplification were classified as undetermined [17]. In this way, we established a workflow that gave reliable results regarding the circulating variants, followed by specific confirmation through SaS.

#### 2.3.2. Seegene Novaplex SARS-CoV-2 Variants VII

**Figure 1:**
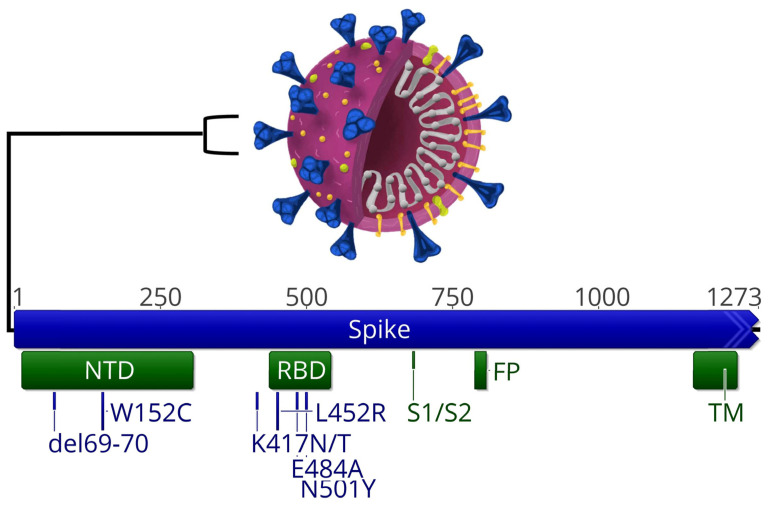
SARS-CoV2 S gene and SNP detected via Novaplex and Allplex assays. SNPs detected are reported in blue color, protein domains where SNPs lay are depicted in green. NTD, N-terminal domain; RDB, receptor-binding domain; TM, transmembrane domain.

The Novaplex SARS-CoV-2 Variants VII Assay (Novaplex) (Seegene, Seoul, Republic of Korea) was carried out according to the manufacturer’s instructions using the CFX96 Real Time PCR Detection System and the BioRad CFX96 Manager software (Bio-Rad, Hercules, CA, USA). The results were analyzed via automated data interpretation using Seegene Viewer software. The Novaplex assay is based on MuDT™ technology, which detects multiple targets with individual Ct values in a single channel without melting curve analysis. By utilizing the change in fluorescence signals between two different temperatures of detection, MuDT™ provides the “real” Ct value of each pathogen even in co-infected cases. MuDT™ allows for the genotyping of four SNP targets in a single tube. The set of primers can detect three SARS-CoV-2 variants of the S gene showing ∆69/70, E484A, and N501Y (Figure 1). The RdRP gene was considered as an internal control. All the NPSs that were only positive for the RdRP gene were classified as positive, but undetermined. Samples showing ∆69/70 deletion (Δ69/70) and N501Y were considered positive for the Alpha variant. Samples with ∆69/70, E484A, and N501Y SNPs were assigned to the Omicron BA.1 variant, while E484A and N501Y SNPs were attributed to the Omicron BA.2 variant.

#### 2.3.3. Seegene Allplex Variants II

To correctly type the Delta variant (B.1.617.2), samples without Δ69/70 were tested with Allplex variants II (Allplex; Seegene Inc., Seoul, Republic of Korea). This assay consists of a multiplex RT-PCR able to detect the S protein mutations W152C, K417N, K417T, and L452R (Figure 1). Samples with the L452R mutation and without Δ69/70 that were negative for N501Y, E484K, W152C, K417T, and K417N were classified as the Delta variant, according to the manufacturer’s instructions. The amplification was carried out in the same instrument used for the Novaplex Variant VII assay (CFX96 Real Time PCR Detection System). Results were analyzed using a dedicated software program, BioRad CFX96 Manager (Bio-Rad, Hercules, CA, USA), which indicates the Ct value for each specific mutation probe. A specimen with a Ct value > 40 was considered negative using both Seegene methods.

### 2.4. Sanger Sequencing

Samples with SGTF underwent SaS of the S gene. Briefly, three fragments of the SARS-CoV-2 S gene (Table 1) were amplified with home-made RT-PCR, followed by SaS. The RT-PCR conditions for M6970 primers were: 52 °C for 30 min; 95 °C for 15 min; 45 cycles at 94 °C for 35 s, 57 °C for 35 s, and 72 °C for 1 min; and a final extension at 72 °C for 10 min. The RT-PCR conditions for VAR1-L and VAR2 primers were: 52 °C for 30 min; 95 °C for 15 min; 45 cycles at 94 °C for 40 s, 60 °C for 50 s, and 72 °C for 1 min; and a final extension at 72 °C for 10 min. Amplification products were sequenced bidirectionally using the 3500XL Genetic analyzer with BigDye Terminator 3.1 chemistry.

### 2.5. Statistical Analysis

The GraphPad v9.0 software (GraphPad, San Diego, CA, USA) was used for the statistical analysis. All results with a *p*-value < 0.005 were considered statistically significant.

The positive per cent agreement (PPA) with SaS, considered as the reference method, was calculated using the formula below:

$$PPA=\frac{\mathrm{Positive}\;\mathrm{samples}\;\mathrm{with}\;\mathrm{Seegene}\;\mathrm{methods}\;\mathrm{or}\;\mathrm{TaqPath}\;\times 100}{\mathrm{Positive}\;\mathrm{samples}\;\mathrm{with}\;\mathrm{Sas}\;\left(\mathrm{true}\;\mathrm{positive}\right)+\mathrm{False}\;\mathrm{negative}\;\mathrm{with}\;\mathrm{Seegene}\;\mathrm{methods}\;\mathrm{or}\;\mathrm{TaqPath}}$$

## 3. Results

**Figure 2:**
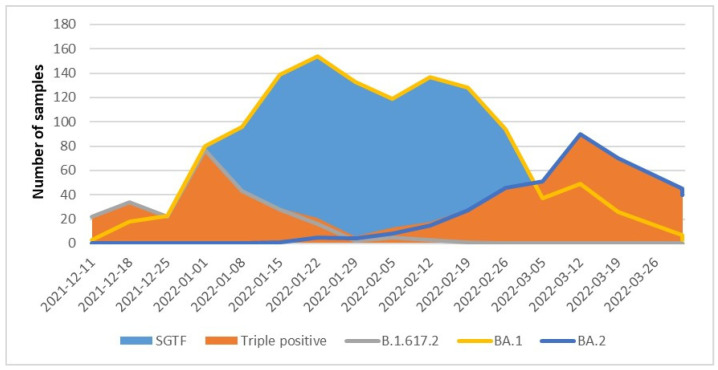
SARS-CoV-2 variants found during the observation period. Variant stacked plot indicating the distribution of SARS-CoV-2 variants among samples collected at INMI from December 2021 to March 2022. Blue and orange areas show results obtained using TaqPath COVID-19 RT-PCR kit; lines show identification of SARS-CoV-2 variants via Sanger sequencing.

At the beginning of the study, the Delta VOC was prevalent, but the Omicron sublineage BA.1 variant became dominant in January 2021, followed by the emergence of the Omicron 2 variant thereafter. Later, with the emergence of the Omicron sublineage BA.2 (characterized by the absence of the Δ69–70 mutation) and the progressive disappearance of Delta variant circulation, the SGTF result was applied to distinguish between Omicron BA.1 and BA.2 lineages (Figure 2) [18].

Overall, from 4 December 2021 to 31 March 2022, *n* = 1350 NPSs positive for SARS-CoV-2 with routine qualitative diagnostic assays were tested using TaqPath, followed by SaS of the S gene. Among these, *n* = 1248 were successfully amplified and classified. For the remaining *n* = 102 (of 1350) samples showing SGTF, the results were inconclusive (undetermined or negative) due to a low viral load, as demonstrated by the high PCR Ct values. Nevertheless, SaS was able to identify SARS-CoV-2 variants in 44 of these samples.

Samples showing the amplification of the complete set of viral genes (S, N, and Orf1ab) were assigned as the Omicron BA.1 variant (*n* = 655); *n* = 251 swabs were identified as the Delta variant and *n* = 342 as Omicron BA.2.

**Figure 3:**
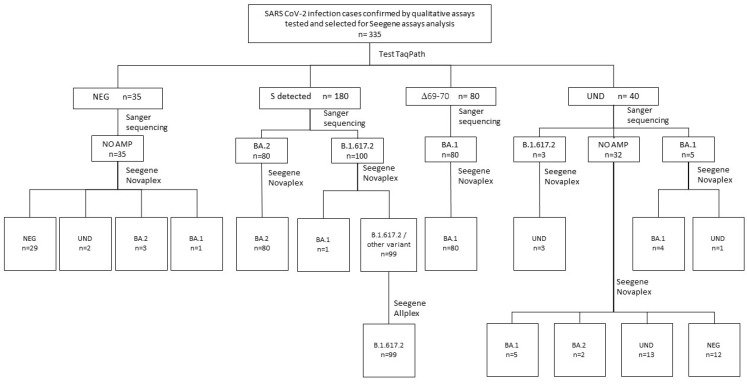
Flow-chart of SARS-CoV-2 variant analysis carried out in SARS-CoV-2-positive NPSs tested with TaqPath. Both SaS and Novaplex assays were used to analyze undetermined and negative TaqPath samples. AllplexVariant II was used to confirm Delta variant. Neg, negative; NO AMP, no amplification; Und, undetermined.

Among the NPSs analyzed with TaqPath representative of different variants and quantitatively available for additional testing, 355 samples were subjected to Novaplex and Allplex assays. TaqPath gave the following results: negative (*n* = 35) in SGTF analysis, but 100% positive with diagnostic qualitative molecular assays; undetermined in SGTF analysis (*n* = 40); Δ69/70-negative and confirmed as the Delta variant via SaS (*n* = 100); Δ69/70-positive and confirmed as Omicron BA.1 via SaS (*n* = 80); Δ69/70-negative and confirmed as Omicron BA.2 via SaS (*n* = 80). All BA.2 samples were collected during February–March 2022, when the Delta variant was disappearing in the Lazio region (Figure 3). These samples underwent Novaplex typing: 27.5% of “undetermined” TaqPath results (*n* = 11 out of 40) and 11.4% of negative TaqPath samples (*n* = 4 out of 35) were identified as different Omicron subtypes (Figure 3).

The Delta variant (B.1.617.2) was only identified via RdRp gene amplification, since Novaplex was developed for Omicron typing (Table S1), giving a “positive” result without typing for other VOCs. To determine if Delta samples were correctly identified as Delta lineages via SaS, the samples were tested with Allplex, providing further confirmation of these data. In fact, 99/100 samples harbored the Delta variant (PPA, 98.0%), while 1 was confirmed to be Omicron BA.1. All PPA values with SaS-typed samples are shown in Table 2.

**Figure 4:**
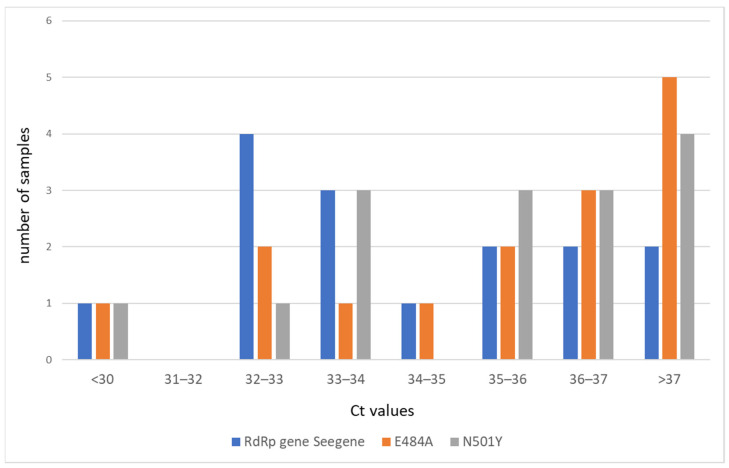
Comparison of Ct values of E484A, N501Y, and RdRp for samples that were undetermined or negative with TaqPath and typed using Novaplex.

The samples that were undetermined with TaqPath showed high ORF1ab and N gene Ct values, ranging from 29.4 to 35.4 (median Ct: 33.3) and from 31.9 to 39.0 (median Ct: 34.2), respectively. The median Ct value for RdRp in samples that were undetermined via TaqPath but typed using Novaplex was 33.2 (ranging from 32.3 to 37.6), while the Ct median value for the E484A mutation was 36.0 (ranging from 32.6 to 37.5), and the Ct median value for the N501Y mutation was 35.6 (ranging from 33.2 to 37.3) (Figure 4; Table S2).

Overall, 17 samples that were undetermined via Novaplex showed Ct values of the RdRp target gene ranging from 32.3 to 36.7 (median Ct: 35.4), resulting in statistically higher values than those observed among samples typed with this assay (median Ct value: 22.0) (*p* < 0.0001, Mann–Whitney Test).

**Figure 5:**
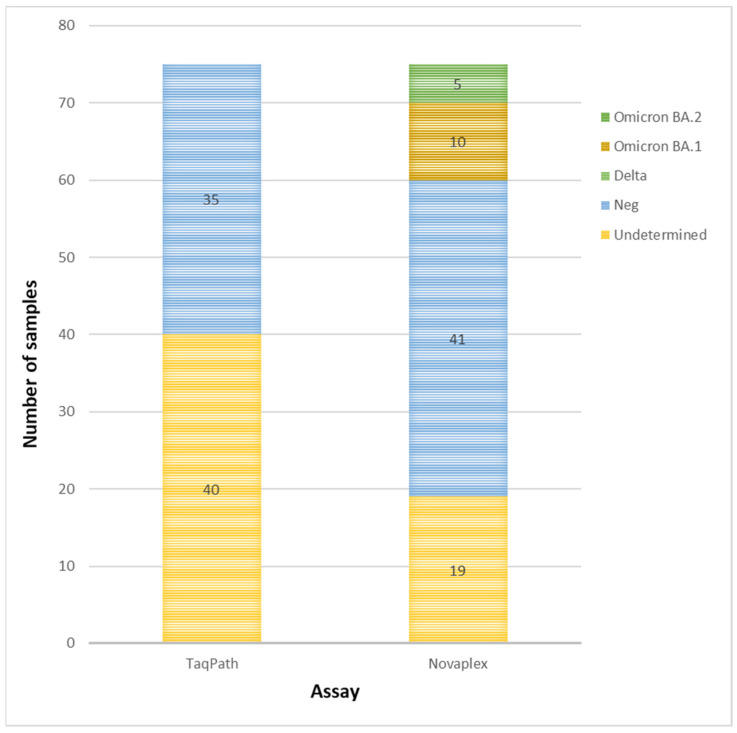
Samples untyped using TaqPath and Novaplex assays. Comparison of number of samples classed as undetermined and negative with both assays.

Among the samples that were classed as negative with the TaqPath assay (*n* = 35), four were typed via Novaplex and were classed as Omicron BA.2, and one was classed as BA.1. Hence, 15/75 (20.0%) samples with inconclusive results (undetermined or negative) with TaqPath were correctly typed using Novaplex (Figure 5). No amplification was revealed via SaS among the 35 negative TaqPath samples.

## 4. Discussion

SARS-CoV-2 has shown high mutation capacity, leading to the spread of new variants in a few months that have had more transmission capacity than the wild-type virus [19,20]. The use of rapid tests to determine new SARS-CoV-2 variants is undoubtedly a challenge in SARS-CoV-2 molecular diagnostics and in tracking the spread of new VOCs. In our study, we compared results obtained with TaqPath with those gained by Novaplex and Allplex, a test employed in variant identification based on SNPs’ detection. In the period of sample collection, the scenario of SARS-CoV-2 variants changed from Delta to Omicron BA.1 to BA.2 prevalence.

Both the TaqPath and Seegene assays are reliable methods in detecting SARS-CoV-2 variants. TaqPath is based on the SGTF method, which allows for the quick identification of Delta from Omicron BA.1 principal lineages through the detection of Δ69/70 mutations in S, without further tests, such as sequencing or confirmation reflex tests. Until the beginning of our observation period (December 2021), Delta variant represented the main VOC circulating in Italy (>80%), while only 5.3% if SARS-CoV-2 strains were BA.1 [21]. Though neither Delta nor Omicron BA.2 have the Δ69/70 deletion, the use of TaqPath as a proxy test gave a reliable indication of Omicron BA.2 presence in samples collected during the February–March 2022 period, when the Delta variant disappeared. Even if the TaqPath proved to be a very sensitive and cost-effective test in the first instance for distinguishing the Delta variant from the Omicron BA.1, the use of SGTFs in the determination of VOCs must be carried out with caution, because there is a risk of misidentifying a non-VOC strain carrying the Δ69/70 mutation, as described in the literature [22], and sequencing analysis is always recommended to confirm the indication of the SGTF assay.

Novaplex successfully identified all Omicron BA.1 and BA.2 variants, and Allplex correctly typed 99/100 Delta VOCs previously determined via SaS of the S region through the R452R SNP. These findings suggest that Novaplex and Allplex are effective diagnostic systems for identifying Omicron BA.1 and BA.2 and Delta variants using multiplex real-time RT-PCR without sequencing. Overall, there was 100% concordance between TaqPath, Novaplex, and SaS in Omicron BA.1 and BA.2 detection, while a sample classified as a Delta variant using SaS was determined to be BA.1 via Allplex. This discordant sample was not repeated due to lack of eluates, so we were unable to assess if there was a sampling error or if the assay was not able to correctly identify this variant. In addition, a discordant finding was reported by another study that compared the performance of two variant assays, TaqPath and COVID-19 CE-IVD Variant Triplex RT-PCR (Biogenex, Fremont, CA, USA): only one sample was discordant in Delta variant detection [23].

The scenario is more complex when samples are classed as negative or undetermined with TaqPath. Among negative samples with TaqPath, four NPSs were typed via Novaplex (one identified as BA.1 and three as BA.2), and two were classed as undetermined. In the undetermined group, 27.5% were correctly typed via Novaplex compared to 20.0% typed via SaS. These results suggest a higher discriminatory power of Novaplex in VOC identification, compared to the Sanger method.

The evolution of the SARS-CoV-2 lineage revealed frequent changes in SNPs over time. An alteration in SNP could compromise the correct screening of variants and the discriminatory power of the assay. With Novaplex, undetermined samples showed a median Ct value of 35.40 for the RdRp target gene, suggesting that these samples were positive but unclassified because of the low viral load and not because of the mutations of the SNPs. Moreover, three samples typed via SaS could not be recognized for the E484A and N501Y SNP. These substitutions are in a highly variable region of the RDB, important due to its involvement in the immune evasion process. Thus, this failure could also be linked to some mutations in flanking regions that hamper the correct binding of probes.

Considering the Ct value of our samples, we were not able to establish a threshold of Ct value above which VOC identification was available. In fact, six samples with Ct > 35 were correctly typed via Novaplex. This assay showed good sensitivity, comparable to that claimed by the ARTIC protocol, which manages to amplify the full genome starting from samples with Ct = 38.1 corresponding to 18 copies/µL [24]. Because the data on SNPs’ performances reported in the literature are contradictory, this method could be used to type samples if SNP fails. Nevertheless, some authors stated that the ARTIC method did not provide adequate coverage in samples with viral loads greater than Ct 30 [25,26]. The reproducibility of results is crucial for a diagnostic test, especially in a pandemic context. In this study, we assessed that TaqPath and Novaplex assays provide reliable and comparable results in samples with SARS-CoV-2 with a Ct value < 33, demonstrating 100% agreement for VOC identification.

## 5. Conclusions

Several RT-PCR assays for VOC identification rely on the detection of specific SNPs [27,28,29], but few data are available regarding their ability to recognize SARS-CoV-2 variants in samples that are classed as undetermined with different assays. Our study, carried out on samples tested with TaqPath, demonstrated the high capability of SNP-based assays to detect VOCs. In fact, in our context, 20% of samples classed as negative or undetermined with TaqPath, among them being twelve not typed via SaS, were instead typed via Novaplex, thus highlighting the higher sensitivity of this method based on SNP multiplex real-time RT-PCR. Although full-genome sequencing remains the main instrument to characterize new strains, diagnostic systems such as Novaplex and Allplex providing VOC identification with rapid turn-around times favor the rapid choice of proper therapy regimens thanks to their powerful approach in diagnostic and clinical settings. The quick recognition of VOCs remains mandatory in surveillance activities and in tracking all changes in the circulating viral population.

## Figures and Tables

**Table 1:** Amino acid coverage of primers used for amplification and sequencing of three regions on SARS-CoV-2 S gene. The main mutations associated with the main VOCs are described.

Primer	5′-3′ Sequence	Amino Acid Coverage	Mutations of Interest
M6970-FW	TGACAAAGTTTTCAGATCCTCAGT	47–171	Del69-70, T95I, G142D, Del143-145, and Del144
M6970-RW	GGTCCATAAGAAAAGGCTGAGA		
VAR1-L FW	TCTCTGCTTTACTAATGTCTATGCAGA	399–616	K417T/N, D428E, N440K, G446S, L452R, Q474R, S477N, T478K, E484A/Q/K, Q493R, G496S, Q498R, N501Y, Y505H, T547K, A570D, and D614G
VAR1-L RW	AACAGGGACTTCTGTGCAGT		
VAR2 FW	GGTTTAACAGGCACAGGTGT	552–722	D614G, H655Y, N679K, P681H/R, and T716I
VAR2 RW	GACACTGGTAGAATTTCTGTGGTA		

**Table 2:** Positive per cent agreement (PPA) of TaqPath, Novaplex and Allplex assays with Sanger sequencing results.

Assay	Sanger Sequencing *n* = 268 *		
	BA.1.617.2 (*n* = 100) PPA	BA.1 (*n* = 85) PPA	BA.2 (*n* = 80) PPA
TaqPath Δ69/70-positive	n.d.	88.90	n.d.
TaqPath Δ69/70-negative	100	n.d.	100
Novaplex	n.d.	100	100
Allplex	98.0	n.d.	n.d.

n.d., not determined; * three samples that were undetermined using TaqPath and typed via Sanger Sequencing as Delta variants were not analyzed using Allplex because template was not available.

## Data Availability

The raw data can be found at the following website: rawdata.inmi.it (accessed on 28 March 2023).

## Supplementary Materials

The following supporting information can be downloaded at: https://www.mdpi.com/article/10.3390/diagnostics13091573/s1, Table S1: Interpretation of Novaplex Variants VII Assay and Allplex Variants II Assay; Table S2: Typing of samples classed as undetermined or negative via TaqPath with Novaplex assay.
