# Supplementary material for: Detection of SARS-CoV-2 Variants via Different Diagnostics Assays Based on Single-Nucleotide Polymorphism Analysis
Source: Diagnostics (Basel). 2023 Apr 27;13(9):1573. doi: 10.3390/diagnostics13091573 (PMC10177602; doi:10.3390/diagnostics13091573)
Supplement: Supplementary file 1 [file diagnostics-13-01573-s001.zip › Specchiarello et al_ TableS1.pdf]

Table S1: Interpretation of Novaplex Variants VII Assay and Allplex Variants II Assay.

Assay	Mutation	Variants of Concern						
		Alpha	Beta	Gamma	Delta	Delta Plus	Omicron	Omicron Stealth
		B.1.1.7	B.1.351	P.1	B.1.617.2	AY.1	B.1.1.529	BA2
Novaplex	Δ69/70	X					X	
	E484A						X	X
	N501Y	X	X	X			X	X
	RdRP	X	X	X	X	X	X	X
Allplex	W152C							
	K417N		X			X	X	X
	K417T			X				
	L452R				X	X		
