# Supplementary material for: Detection of SARS-CoV-2 Variants via Different Diagnostics Assays Based on Single-Nucleotide Polymorphism Analysis
Source: Diagnostics (Basel). 2023 Apr 27;13(9):1573. doi: 10.3390/diagnostics13091573 (PMC10177602; doi:10.3390/diagnostics13091573)
Supplement: Supplementary file 1 [file diagnostics-13-01573-s001.zip › Specchiarello et al_ TableS2.pdf]

Table S2: Typing of samples resulted undetermined or negative by TaqPath with Novaplex Variant VII assay.

Sample code	TaqPath Results	RdRp gene	Δ69/70	E484A	N501Y	Novaplex output
13	Undetermined	32.45	31.81	32.64	33.24	Omi B.1.1.529
30	Undetermined	32.31	32.78	34.01	33.82	Omi B.1.1.529
34	Undetermined	32.86	33.12	37.25	36.94	Omi B.1.1.529
42	Undetermined	32.30	32.83	33.20	33.37	Omi B.1.1.529
43	Undetermined	37.69	35.66	36.53	36.26	Omi B.1.1.529
58	Undetermined	35.95	35.95	36.79	36.4	Omi B.1.1.529
64	Undetermined	36.33	33.99	36.09	35.68	Omi B.1.1.529
78	Undetermined	35.22	35.45	37.46	37.07	Omi B.1.1.529
79	Undetermined	33.26	N/A	35.21	35.04	Omi BA.2
89	Undetermined	33.66	N/A	37.51	37.36	Omi BA.2
96	Undetermined	33.16	33.47	35.80	35.44	Omi B.1.1.529
12	NO_AMP	37.67	N/A	32.91	32.99	Omi BA.2
53	NO_AMP	36.49	N/A	37.52	37.18	Omi BA.2
73	NO_AMP	34.95	N/A	37.72	37.44	Omi BA.2
83	NO_AMP	25.16	24.57	26.43	27.07	Omi B.1.1.529

NO_AMP, no amplification; Omi, Omicron;
